# High Expression of Integrin α3 Predicts Poor Prognosis and Promotes Tumor Metastasis and Angiogenesis by Activating the c-Src/Extracellular Signal-Regulated Protein Kinase/Focal Adhesion Kinase Signaling Pathway in Cervical Cancer

**DOI:** 10.3389/fonc.2020.00036

**Published:** 2020-02-14

**Authors:** Qiqiao Du, Wei Wang, Tianyu Liu, Chunliang Shang, Jiaming Huang, Yuandong Liao, Shuhang Qin, Yili Chen, Pan Liu, Junxiu Liu, Shuzhong Yao

**Affiliations:** ^1^Department of Obstetrics and Gynecology, The First Affiliated Hospital of Sun Yat-sen University, Guangzhou, China; ^2^Department of Obstetrics and Gynecology, Peking University Third Hospital, Beijing, China

**Keywords:** cervical cancer, metastasis, integrin α3, angiogenesis, focal adhesion kinase

## Abstract

**Background:** Cervical cancer remains a leading cause of death in women due to metastasis to distant tissues and organs. Integrins are involved in cancer metastasis. However, whether integrin α3 participates in cervical cancer metastasis is under investigation. In this study, we explored the effect and detailed mechanism through which integrin α3 regulates cervical cell migration, invasion, and angiogenesis.

**Methods:** First, we explored the mRNA and protein expression levels of integrin α3 in cervical cancer cell lines and tissue samples obtained from patients. After knocking down the expression of integrin α3 using shRNA, the proliferation, migration, and invasion of cervical cancer cells, as well as the possible signaling pathways involved, were investigated *in vitro*. In addition, tube formation, proliferation, and migration of human umbilical vein endothelial cells were tested to identify their effect on angiogenesis. Zebrafish tumor migration and nude mouse lung metastasis models were utilized for the *in vivo* analysis.

**Results:** We examined samples from 142 patients with cervical cancer and 20 normal cervixes. Integrin α3 was highly expressed in patients and predicted poor overall survival and disease-free survival. In SiHa cells, treatment with integrin α3 shRNA induced the phosphorylation of protein focal adhesion kinase and enhanced focal adhesion. These events were mediated by the activation of c-Src and extracellular signal-regulated protein kinase cascades. Consequently, integrin α3 increased the migratory ability of SiHa cells. In addition, knockdown of integrin α3 decreased the tube formation, proliferation, and migration of human umbilical vein endothelial cells, as well as the levels of matrix metalloproteinase-9, indicating its effect on angiogenesis. Stable transfection with integrin α3 shRNA reduced the migratory ability of SiHa cells in the zebrafish model and diminished lung metastasis in the xenograft mouse model.

**Conclusion:** Integrin α3 recruits the c-Src/extracellular signal-regulated protein kinase cascade, leading to phosphorylation of focal adhesion kinase. Moreover, it regulates focal adhesion, endowing cervical cancer cells with potentiated migratory and invasive ability, and promotes angiogenesis via matrix metalloproteinase-9. Our findings may shed light on the mechanism involved in cervical cancer metastasis and highlight integrin α3 as a candidate prognostic biomarker and therapeutic target in patients with cervical cancer.

## Introduction

Cervical cancer (CC) is one of the most common gynecologic malignancy. Despite efficient vaccination and screening, it ranks fourth among the most frequently diagnosed types of cancer and is a leading cause of cancer-related death in women worldwide (1). The primary reason for CC-related mortality is the metastasis of cancer cells to distant or nearby tissues and organs (2). Metastasis to the lymph nodes or other parts of the body is associated with markedly worse prognosis for patients with CC (3). Metastasis is a complex, multistep process that includes invasion, dissemination, survival in the circulation, arrest at a distant organ site, and finally metastatic colonization (4). Therefore, it is urgent and vital to investigate the mechanism involved in CC metastasis for its prevention and treatment. However, thus far, the molecular mechanism underlying CC metastasis remains largely unknown. Hence, the identification of biomarkers of early prognosis and metastasis and the discovery of novel treatment targets to improve survival are of great importance.

Integrins form a molecular family characterized as the cell surface receptors responsible for anchoring cells to the extracellular matrix. Recently, they were discovered to participate in the dynamic processes in tumor cells, such as proliferation, migration, and invasion, which may lead to cancer cell survival and dissemination (5). Integrins show strong correlation with CC (6). As an important member of the integrin family, the role of integrin α3 in the pathological process of cancer has been determined. In addition, its association with cancer metastasis has been identified in several studies. Integrin α3 acts as pro-tumoral or tumoricidal factor in various types of cancer. For instance, integrin α3 suppresses the metastasis of prostate cancer (7), whereas its overexpression predicts poor survival and is associated with distant metastasis in nasopharyngeal carcinoma (8). Thus, it is of great importance to discover its role in the context of specific types of cancer. However, whether integrin α3 can affect CC metastasis or cell migration and invasion remains unclear and warrants investigation.

Integrins function as cell surface adhesion receptors, which connect the extracellular matrix to the cytoskeleton and activate adhesion-dependent intracellular signaling pathways (9). Notably, integrins can activate numerous signaling intermediates, such as focal adhesion kinase (FAK), Src, mitogen-activated protein kinase, extracellular signal-regulated protein kinase (ERK), mammalian target of rapamycin (mTOR) (10, 11). Among these intermediates, FAK has attracted considerable attention owing to its key regulatory role in cancer migration and metastasis. It has been reported that integrin α3 upregulates the phosphorylation of FAK in hepatocellular carcinoma, indicating that FAK may be the driving force for the oncogenic activity of integrin α3 (12). Another functional aspect of the effect of integrins that attracts interest is the regulation of angiogenesis, which is involved in tumorigenesis and the metastatic process (13). In addition, integrin α3 also modulates the crosstalk between keratinocytes and endothelial cells to induce angiogenesis (14).

In this study, we identified integrin α3 as a prognostic factor in CC. We showed that high expression of integrin α3 predicted poor prognostic outcome in patients with CC. *In vitro* and *in vivo* assays revealed that integrin α3 activates the migration and invasion of CC cells via activation of the c-Src/Erk/FAK signaling pathway. In addition, integrin α3 exerts an effect on angiogenesis via matrix metalloproteinase-9 (MMP-9), which is also involved in CC metastasis. Our data identified integrin α3 as an oncogenic factor, which can promote CC metastasis and angiogenesis via the c-Src/Erk/FAK signaling pathway. Modulation of this pathway or identification of this prognostic factor in patients may provide a therapeutic option against CC metastasis.

## Materials and Methods

### Cell Cultures and Treatments

Human CC cell lines HeLa, C33A, SiHa, ME180, and MS751 were obtained from Shanghai Institutes for Biological Sciences (Chinese Academy of Sciences, Shanghai City, China) and incubated in Dulbecco's modified Eagle's medium/F-12 or Dulbecco's modified Eagle's medium containing 10% fetal bovine serum, 0.2 UI/ml of insulin, l-glutamine, and penicillin streptomycin under a 5% carbon dioxide atmosphere at 37°C. Human umbilical vein endothelial cells (HUVECs) were cultured as previously described (15).

### RNA Isolation and Quantitative Real-Time Polymerase Chain Reaction

Total RNA was isolated using RNAiso plus reagent (TAKARA, Dalian, China) and reverse-transcribed into cDNA using PrimeScript RT Master Mix (TAKARA, Dalian, China) according to the protocol provided by the manufacturer. Quantitative real-time (qRT)-PCR analyses were performed utilizing SYBR Premix Ex Taq (TAKARA, Dalian, China). In the PCR cycling (40 cycles), pre-denaturation was accomplished in 30 s at 95°C, whereas the parameters for denaturation and annealing were set at 95°C for 5 s and 60°C for 34 s, respectively. The qRT-PCR primer sequences of integrin α3 were as follows (16): forward, 5′- GCTGTATCCCACGGAGATCA-3′ and reverse, 5′- GTCAGCCTCTCTGTCTCTGA-3′. The qRT-PCR was repeated at least thrice. Relative fold changes in expression were calculated using the comparative cycle threshold (2^−ΔΔCt^) method. Expression data were normalized to the geometric mean with reference to the housekeeping gene β-actin.

### Immunoblotting

Cell lysates were separated using sodium dodecyl sulfate–polyacrylamide gel electrophoresis. The antibodies used were as follows: anti-phospho-Y397-Fak (ab81298) and anti-FAK (ab131435) from Abcam (Cambridge, MA, USA); anti-Tyr416-phosphor-c-Src (2010s), anti-c-Src (2108s), anti-phosphoERK1/2 (T202/Y204), and anti-ERK1/2 (4370s) from Cell Signaling Technology (Danvers, MA, USA); anti-integrin α3 (21992-1-AP) from Proteintech (Rosemont, IL, USA); and β-actin (sc-81178) from Santa Cruz (Santa Cruz, CA, USA). Primary and secondary antibodies were incubated with the polyvinylidene difluoride membranes using standard techniques. Immunodetection was accomplished using enhanced chemiluminescence. Chemiluminescence was measured with a quantitative digital imaging system (Quantity One; BioRad, Hercules, CA, USA) allowing to check for saturation. Overall emitted photons were quantified for each band, particularly for loading controls, which were homogeneously loaded.

### Cell Immunofluorescence

SiHa cells were seeded on coverslips and exposed to treatments. Cells were fixed with 4% paraformaldehyde for 30 min and permeabilized with 0.1% Triton X for 5 min. Blocking was performed with 3% bovine serum albumin for 20 min. Cells were incubated with antibodies against responding primary antibody (anti-Ki67, ab15580; Abcam) and linked to Alexa Fluor^®^ 488 or 555 (Cell Signaling Technology). After being washed, the nuclei were counterstained with 4′-6-diamidino-2-phenylindole (DAPI; Sigma-Aldrich, St. Louis, MO, USA). The coverslips were mounted using Vectashield mounting medium (Vector Laboratories, Burlingame, CA, USA). Immunofluorescence was visualized using an Olympus BX41 microscope (Tokyo, Japan).

### Transfection Experiments

SiHa cells were transfected with siRNA or plasmids using Lipofectamine (Thermo Fisher Scientific, St. Louis, MO, USA) according to the protocol provided by the manufacturer. The shRNA, siRNA, plasmids, and lentivirus used in this study were listed as pLKD-CMV-R&PR-U6-shRNA-ITGA3/corresponding control shRNA (520137HN; Obio Technology, Shanghai City, China), FAK siRNA (6568; Cell Signaling Technology), pcDNA3-c-Src plasmid, pcDNA3-ERK2 plasmid (42202/8974; Addgene, Cambridge, MA, USA), and ITGA3 plasmid (VH878515, Vigenebio, Jinan City, China). Cells (60% confluent) were serum starved for 8 h followed by incubation with target siRNA/shRNA/plasmid or corresponding control for 12 h in Opti-MEM Reduced Serum Media (Thermo Fisher Scientific). The serum-containing media (10% fetal bovine serum) were subsequently added for 36 h prior to conducting the experiments and/or functional assays. Silencing of target proteins was assessed through protein analysis up to 48 h after transfection. Lentivirus lenti-ITGA3 (630189HN; Obio Technology) was transfected into SiHa cells. Stable cell lines were selected for 10 days with 0.5 mg/ml of puromycin 48 h after infection.

### MTT Assay

SiHa cells were seeded into 96-well plates with 5 × 10^3^ cells per well and 100 μl of culture medium and incubated for 24 h. Following transfection with shRNA for 48 h, the medium was replaced with serum-free medium, and MTT labeling reagent (10 μl, final concentration 0.5 mg/ml, MTT assay kit, ab211091; Abcam) was added. The microplate was subsequently incubated for 3 h in humidified atmosphere. MTT solvent was added, the microplate was shaken on an orbital shaker for 15 min, and absorbance was measured at OD590 nm.

### Wound Healing Assay

SiHa cells were seeded into six-well plates and incubated for 24–48 h. Following transfection, the cell confluency was approximately 100%. A pipette tip was used to perform a scratch wound area (multiple lines in a well). The wells were washed twice with phosphate-buffered saline. The medium was replaced with normal medium, and the plates were observed every 12 h. The observation ended 48 h after performing the scratch wound.

### Transwell Migration/Invasion Assays

For the transwell migration assay, 2 × 10^4^ cells were seeded in the upper chamber of the 8-μm transwell inserts (BD Biosciences, Franklin Lakes, NJ, USA) with 100 μl of serum-free medium. Medium (500 μl) containing 10% bovine serum albumin was added to the lower chamber. After 24 h of incubation, cells in the upper chamber were carefully removed. The cells adhering to the membrane were fixed in methanol for 15 min and stained with 0.1% crystal violet (KeyGEN Biotech, Nanjing City, China) for 30 min. For the invasion assays, the upper chamber was precoated with 50 μl of Matrigel (BD Biosciences, Bedford, MD, USA) diluted 1:4 with serum-free medium, and 2 × 10^5^ cells in 100 μl of serum-free medium were seeded. The rest of the procedure was similar to that of the transwell migration assay.

### Real-Time Migration/Invasion Monitoring

The invasion assays were performed in CIM-16 plates with 8-μm pore membranes (5665817001, ACEA Biosciences, San Diego, USA). Wells were coated with (invasion) or without (migration) 20 μl of 25% Matrigel (#356234; BD Biosciences, Shanghai City, China) and allowed to gel at 37°C, 5% carbon dioxide for 4 h. Subsequently, the wells of the bottom chamber were filled with 160 μl of medium containing 10% serum, whereas those of the top chamber were filled with 30 μl of medium without serum. Subsequently, the top and bottom chambers were assembled together. The assembled CIM-16 plate was equilibrated for 1 h at 37°C, 5% carbon dioxide. SiHa cells (2 × 10^4^ cells per well) in 100 μl of medium were seeded onto the top chambers of CIM-16 plates and placed into the xCELLigence system (ACEA Biosciences, San Diego, USA) for data collection after a 30-min incubation at room temperature. The xCELLigence software was used to collect impendence data (reported as cell index) at least once every hour.

### Tube Formation Assay

HUVEC suspension was added to well of a 96-well BD BioCoat angiogenesis plate (BD Biosciences) that had been precoated with Matrigel matrix. After treatment, cells were stained with calcein AM (10 mM). The tube formation was visualized and pictured under Olympus BX41 microscope. The tube-like network was traced, and total length was quantified via Wimtube formation module in WIMASIS Image Analysis (Munich, Germany).

### Enzyme-Linked Immunosorbent Assay

ELISA kit (ab100610, Abcam) was used to detect MMP-9. Samples were processed according to the manufacturer's instructions. Cell culture supernatants were collected after treatment. Absorbance was measured at 450 nm with a microplate reader (Thermo Fisher Scientific, Waltham, MA, USA).

### Xenograft Mouse Metastatic Model

Mice were bred under specific pathogen-free (SPF) conditions in the Department of Sun Yat-sen University Animal Center, as approved by the China Care Committee Institute. The female BALB/c nude mice (4–6 weeks of age, 18–20 g) were randomly divided into two groups (*n* = 5/group). Xenograft mouse metastatic model was utilized for comparing the metastatic ability between different stable cell lines (Sh-Ctrl vs. Sh-integrin α3). The cells (2 × 10^6^/100 μl per mouse) were injected intravenously into the tail vein of female BALB/c-nu mice. The mice were sacrificed, and the lungs were removed 6 weeks later and fixed with 4% paraformaldehyde for hematoxylin and eosin (H&E) staining. The number of visible tumor nodules and mice pulmonary metastatic foci was recorded and confirmed by specialized pathologists.

### Zebrafish Tumor Model

All animal studies were approved by the Animal Ethical and Welfare Committee of Sun Yat-sen University (Guangzhou City, China). Zebrafish embryos were raised at 28°C under standard experimental conditions. Zebrafish embryos at the age of 24 hpf were incubated in aquarium water containing 0.2 mmol/l of 1-phenyl-2-thio-urea (#P7629, Sigma-Aldrich, Darmstadt, Germany). At 48 hpf, zebrafish embryos were dechorionated with a pair of sharp-tip forceps and anesthetized with 0.04 mg/ml of tricaine (MS-222, Sigma-Aldrich). Anesthetized embryos were subjected to microinjection. Stable cell lines were established through transfection with a lentivirus. Approximately 500 cells were resuspended in Dulbecco's modified Eagle's medium (SH30081.02, Hyclone, USA), and 5 nl of the cell solution was injected into the perivitelline space of each embryo using an Eppendorf microinjector (FemtoJet 5247, Eppendorf and Manipulator MM33-Right; Märzhäuser Wetzlar, Wetzlar, Germany). Zebrafish embryos were monitored for 72 h to investigate tumor migration using a fluorescent microscope.

### Immunoprecipitation Assays

SiHa cells were lysed in 100 mM of Tris-hydrochloride, pH 6.8, 4% sodium dodecyl sulfate, 20% glycerol, 1 mM of sodium orthovanadate, 1 mM of sodium fluoride, and 1 mM of phenylmethylsulfonyl fluoride. Equal amounts of cell lysates were incubated with 4 μg of precipitating antibody against integrin α3 or c-Src for 1 h at room temperature under gentle agitation. Subsequently, 1:1 protein A-agarose slurry (50 μl) was added, and the samples were rolled at room temperature for another hour. The samples were then pelleted, washed, and resuspended in 20 μl of elution buffer for immunoblotting.

### Immunohistochemistry

Immunohistochemistry (IHC) staining was performed in paraffin-embedded tissue samples cut in 4-cm sections. Slides were deparaffinized in xylene, rehydrated using a series of graded alcohols, and blocked with 10% goat serum prior to incubation with a primary antibody overnight. This was followed by incubation with a horseradish peroxidase-conjugated secondary antibody for 30 min at room temperature. Antibody binding was detected using 3,3′-diaminobenzidine, and the reaction was terminated through immersion of tissue sections in distilled water, following the appearance of brown color. Tissue sections were counterstained using hematoxylin, dehydrated in graded ethanol, and mounted. For the statistical analysis, the IHC scores (range: 0–6) were evaluated; and the staining score of 4 was defined as the cutoff value, as previously described Wang et al. (17). Thus, patients with different positive levels of expression were divided into low- and high-staining groups.

### Clinical Specimens

For IHC staining, a total of 142 paraffin-embedded tissues of CC collected from January 2006 to December 2012 were obtained from the archives of the Department of Pathology at the First Affiliated Hospital of Sun Yat-sen University. All enrolled patients with CC were matched from stage Ia2–IIa2 with available follow-up data, and they underwent radical hysterectomy and lymphadenectomy. None of the patients were treated with radiotherapy or chemotherapy prior to surgery. Informed consent was provided by each patient. The 20 normal uterine cervical tissues (controls) were collected from female patients who underwent hysterectomy for non-malignant conditions. The study was approved by the Ethics Committee of the First Affiliated Hospital of Sun Yat-sen University.

### Statistical Analysis

Statistical analyses were performed using the SPSS version 20.0 statistical software (IBM Corp., Armonk, NY, USA). All values are expressed as mean ± standard deviation. Statistical differences between the mean values were determined through analysis of variance, followed by Fisher's protected least significance difference. The χ^2^ test and Fisher's exact test were used to analyze the relationship between integrin α3 expression and the clinicopathological characteristics. Survival curves were plotted via the Kaplan–Meier method and compared using the log-rank test. In all cases, *P* < 0.05 denoted statistical significance.

## Results

### Integrin α3 Is Upregulated in Human Cervical Cancer Tissues and Cell Lines

**Figure 1:**
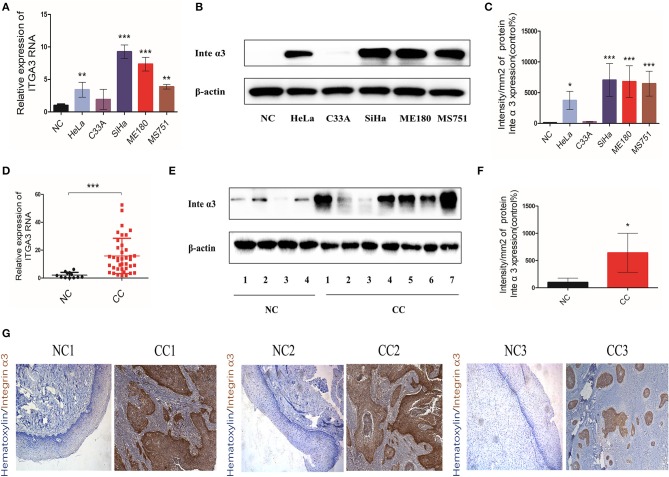
Integrin α3 is overexpressed in cervical cancer tissue and cell lines. **(A)** The results of the quantitative real-time (qRT)-PCR analysis showed the mRNA levels of ITGA3 expression in different cervical cancer cell lines. Normal cervix (NC) tissue was used as the normal control. **(B)** Western blotting was applied to show the protein levels of integrin α3 in different cervical cancer lines. **(C)** Quantification results of the protein intensity of **(B)**. **(D)** Results of the qRT-PCR analysis of ITGA3 expression in normal cervix tissue (*n* = 12) and cervical cancer (CC) tissue (*n* = 39). **(E)** Western blotting was used to analyze the protein expression of integrin α3 in NC (*n* = 4) and CC (*n* = 7). **(F)** Quantification results of the protein intensity of **(E)**. **(G)** Immunohistochemistry (IHC) assays were performed to investigate the protein expression of integrin α3 in three trial pairs of NC and CC. Original magnification: ×100. ^*^P < 0.05 vs. corresponding control; ^**^P < 0.01; ^***^P < 0.001.

We detected the mRNA and protein expression levels of integrin α3 in human CC tissues and cell lines to explore its role in CC. Western blotting and qRT-PCR were performed in five CC lines (i.e., HeLa, C33A, SiHa, ME180, and MS751). Four cell lines, including HeLa, SiHa, ME180, and MS751, had higher mRNA expression levels of integrin α3 than the normal cervix (NC) (Figure 1A). Consistently, these four cell lines also expressed higher levels of integrin α3 protein (Figures 1B,C).

The RNA of 12 NC tissues and 39 CC tissues was isolated to determine whether integrin α3 is also upregulated in human CC tissues. In addition, western blotting was performed in four NC samples and seven CC samples. The results showed higher expression levels of integrin α3 in CC tissues vs. NC tissues (Figures 1D–F). Moreover, three pairs of CC tissues and matched NC tissues were selected for IHC analysis. The CC foci showed strong positive staining for integrin α3 (Figure 1G).

Collectively, these data suggested that integrin α3 was aberrantly upregulated (both mRNA and protein levels) in CC tissues and cell lines, which triggered our interest to further explore its role in CC.

### High Expression of Integrin α3 Is Related to Poor Clinicopathological Features and Predicts Poor Prognosis

**Figure 2:**
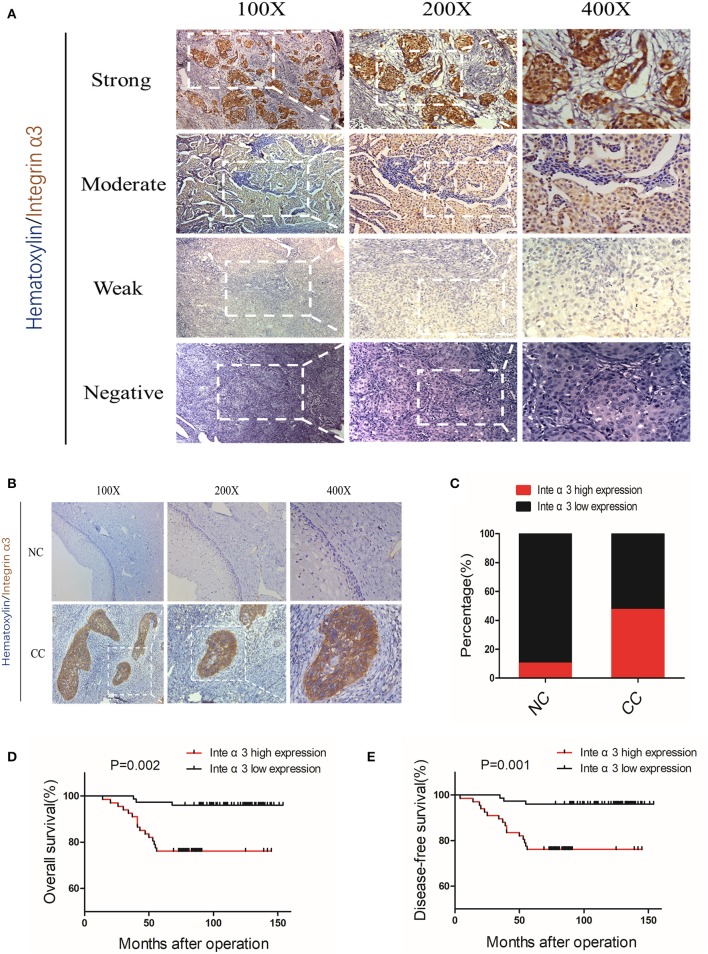
Integrin α3 is frequently upregulated in cervical cancer tissue and significantly associated with overall survival and disease-free survival. **(A)** Representative immunohistochemistry (IHC) image of cervical cancer (CC) tissue with different staining intensities of integrin α3. **(B)** Representative IHC image of CC tissue of classic positive staining of integrin α3 under different magnifications (×100, ×200, and ×400). **(C)** Bar graph presenting the statistical composition of high/low expression in normal cervix (NC) and CC tissues. **(D,E)** Overall survival and disease-free survival of patients with high or low integrin α3 expression, respectively. The survival curve was constructed using the log-rank test. The *P*-value is shown in each panel.

The IHC analysis of three pairs of pre-experimental CC–NC tissues showed that integrin α3 was more likely to be upregulated in CC tissues. Subsequently, we enlarged the examined tissue groups to 20 NC tissues and 142 CC tissues for further analysis. The representative images of the scoring system for negative, weak, moderate, and strong staining are shown in Figure 2A; classic images of integrin α3 expression in CC foci are shown in Figure 2B. Notably, the ratio of high integrin α3 expression was markedly higher in the CC group than in the NC group (47.18 vs. 10%, respectively) (Figure 2C).

According to the expression of integrin α3 in CC tissues, the patients were divided into high or low expression groups. The relationship between integrin α3 expression and clinicopathological features was analyzed; high expression of integrin α3 was significantly related to several poor features, including differentiation grade (*P* = 0.014), recurrence (*P* < 0.001), and vital status at follow-up (*P* < 0.001) (Table 1). Otherwise, there was no marked relationship found between the expression and age, International Federation of Gynecology and Obstetrics (FIGO) stage, tumor size, pathological types, stromal invasion, lymph vascular space invasion, pelvic lymph node metastasis, vaginal involvement, or parametrial infiltration (Table 1).

**Table 1:** Correlation between integrin α3 expression and clinicopathological characteristics of patients with cervical cancer.

**Clinicopathological variable**		**Integrin α3**		***P*-value**
	**Total (*n* = 142)**	**Low expression**	**High expression**	
**AGE, YEARS**				
≤42	55	33	22	
>42	87	42	45	0.174
**FIGO STAGE**				
Ia2	8	3	5	
Ib1	99	55	44	
Ib2	10	4	6	
IIa1	16	6	10	
IIa2	9	7	2	0.960
**TUMOR SIZE**				
≤4	114	62	52	
>4	28	13	15	0.451
**PATHOLOGIC TYPE**				
Squamous cell carcinoma	113	58	55	
Adenocarcinoma	20	12	8	
Adenosquamous carcinoma	9	5	4	0.501
**STROMAL INVASION**				
<1/2	55	26	29	
≥1/2	87	49	38	0.294
**LYMPHOVASCULAR SPACE INVASION**				
Yes	16	10	6	
No	126	65	61	0.412
**DIFFERENTIATION GRADE**				
Well	11	3	8	
Moderate	51	23	28	
Poor	80	49	31	0.014^*^
**PELVIC LYMPH NODE METASTASIS**				
Yes	29	18	11	
No	113	57	56	0.265
**VAGINAL INVOLVEMENT**				
Yes	3	3	0	
No	139	72	67	0.099
**PARAMETRIAL INFILTRATION**				
Yes	2	2	0	
No	140	73	67	0.180
**RECURRENCE**				
Yes	13	2	11	
No	129	73	56	0.005^**^
**VITAL STATUS AT FOLLOW-UP**				
Alive	123	72	51	
Expired	19	3	16	0.002^**^

*FIGO, International Federation of Gynecology and Obstetrics*.

* *P < 0.05*.

** *P < 0.01*.

*Bold values represent p < 0.05*.

Univariate and multivariate analyses were performed to determine whether integrin α3 is an independent risk factor. Our data revealed that pelvic lymph node metastasis (*P* = 0.025), recurrence (*P* < 0.001), and integrin α3 expression (*P* = 0.016) were the independent prognostic factors for overall survival (OS) (Table 2). Similarly, pelvic lymph node metastasis (*P* = 0.02), recurrence (*P* < 0.001), and integrin α3 expression (*P* = 0.019) were also identified as the independent prognostic factors for disease-free survival (DFS) (Table 2). Furthermore, Kaplan–Meier survival curves and log-rank test survival analysis were performed according to the expression of integrin α3 in patients with CC, demonstrating that the high expression group had shorter OS and DFS than had the low expression group (*P* = 0.002 and *P* < 0.001, respectively) (Figures 2D,E).

**Table 2:** Univariate and multivariate analyses of factors associated with overall survival and disease-free survival.

**Clinicopathological variable**		**Overall survival**			**Disease-free survival**		
		**Univariate**	**Multivariate**		**Univariate**	**Multivariate**	
	**Total (*n* = 142)**	***P*-value**	**RR (95% CI)**	***P*-value**	***P*-value**	**RR (95% CI)**	***P*-value**
**AGE, YEARS**							
≤42	55						
>42	87	0.399	N	N	0.393	N	N
**FIGO STAGE**							
Ia2	8						
Ib1	99						
Ib2	10						
IIa1	16						
IIa2	9	0.630	N	N	0.631	N	N
**TUMOR SIZE**							
≤4	114						
>4	28	0.262	N	N	0.268	N	N
**PATHOLOGIC TYPES**							
Squamous cell carcinoma	113						
Adenocarcinoma	20						
Adenosquamous carcinoma	9	0.332	N	N	0.336	N	N
**STROMAL INVASION**							
<1/2	55						
≥1/2	87	0.443	N	N	0.437	N	N
**LYMPHOVASCULAR SPACE INVASION**							
Yes	16						
No	126	<0.001^*^	0.905 (0.215–3.811)	0.892	<0.001^*^	1.179 (0.297–4.686)	0.815
**DIFFERENTIATION GRADE**							
Well	11						
Moderate	51						
Poor	80	0.414	N	N	0.409	N	N
**PELVIC LYMPH NODE METASTASIS**							
Yes	29						
No	113	<0.001^*^	0.240 (0.069–0836)	0.025^*^	<0.001^*^	0.234 (0.069–0.794)	0.020^*^
**VAGINAL INVOLVEMENT**							
Yes	3						
No	139	0.530	N	N	0.529	N	N
**PARAMETRIAL INFILTRATION**							
Yes	2						
No	140	0.610	N	N	0.609	N	N
**RECURRENCE**							
Yes	13						
No	129	<0.001^*^	0.009 (0.002–0.043)	<0.001^*^	<0.001^*^	0.007 (0.001–0.037)	<0.001^*^
**INTEGRIN α3 EXPRESSION**							
Low	75						
High	67	0.002^*^	0.173 (0.042–0.721)	0.016^*^	0.002^*^	0.184 (0.044–0.760)	0.019^*^

*RR, relative risk; CI, confidence interval; N, not applicable; FIGO, International Federation of Gynecology and Obstetrics*.

* *P < 0.05*.

*Bold values represent p < 0.05*.

Conclusively, these data showed that high expression of integrin α3 was correlated with several clinicopathological features. More importantly, integrin α3 could be used as the independent prognostic factor and may serve as a novel biomarker for patients with CC.

### Integrin α3 Promotes Migration and Invasion of Cervical Cancer Cells, but Not Proliferation *in vitro*

**Figure 3:**
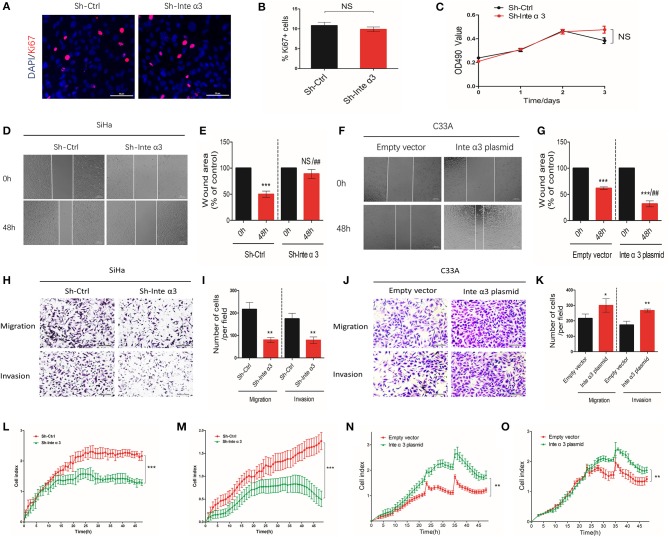
Integrin α3 promotes the migration and invasion, but not proliferation, of cervical cancer cells *in vitro*. **(A)** SiHa cells were treated with control shRNA (Sh-Ctrl) and integrin α3-shRNA (Sh-Inte α3). Subsequently, immunofluorescence staining of cells with Ki-67 was performed to investigate cell proliferation. **(B)** Quantification of Ki-67-positive cells per field in **(A)** (original magnification: ×100). **(C)** The MTT assay was used to analyze the proliferation rate between the Sh-Ctrl and Sh-Inte α3 groups. **(D)** The wound healing assay was applied in SiHa cells to explore the effect of integrin α3 on cell migration *in vitro* in the Sh-Ctrl and Sh-Inte α3 groups, respectively. **(E)** Quantification of the wound area in **(D)**. **(F)** The wound healing assay was applied in C33A cells to explore the effect of integrin α3 on cell migration *in vitro* in the empty vector and Inte α3 plasmid groups. **(G)** Quantification of the wound area in **(F)**. **(H–K)** Transwell migration and invasion assays were performed to investigate the effect of integrin α3 on the migration and invasion of cervical cancer cells either knocking down in SiHa or overexpressing in C33A. Quantification results of the migration and invasion of cells per field in **(I,K)**. **(L,M)** Migration and invasion were tested in the Sh-Ctrl and Sh-Inte α3 groups through real-time migration/invasion monitoring. **(N,O)** Migration and invasion were tested in empty vector and Inte α3 plasmid groups through real-time migration/invasion monitoring. All experiments were repeated thrice with consistent results, and the representative images are shown. ^*^P < 0.05 vs. corresponding control; ^**^P < 0.01; ^***^P < 0.001; ^*##*^*P* < 0.01.

On the basis of the aberrant expression of integrin α3 observed in CC tissues and cell lines, we decided to explore its effect on the pathological process in CC cells, including cell proliferation, migration, and invasion. We particularly selected the SiHa cell line for further *in vitro* knocking down assays, as its integrin α3 expression remains one of the highest among CC cell lines (Figures 1A–C), while we also chose the C33A cell line for overexpression assays owing to its lowest integrin α3 expression among those cell lines. MTT assay and Ki-67 immunofluorescence staining were performed to uncover its role in cell proliferation. However, following the knockdown of integrin α3 expression using shRNA (Figures S1B,C), the proliferation of SiHa cells was unchanged (Figures 3A–C).

For the detection of cell migration and invasion, we performed wound healing assays, transwell migration/invasion assays, and real-time monitoring of migration/invasion. Fewer wound healing areas were noticed in the integrin α3-knockdown group (Sh-Inte α3 group) (Figures 3D,E), indicating that knocking down of integrin α3 could reduce the migration of CC cells. To the contrary, overexpressing the integrin α3 (Figures S1D,E) in C33A cell could lead to more would healing areas (Figures 3F,G). Moreover, the results of the transwell migration/invasion assays showed that fewer cells migrated or invaded through the chamber in the Sh-Inte α3 group vs. the control group (Sh-Ctrl group) (Figures 3H,I) and more migrated or invaded cells in the Inte α3 plasmid group compared with the empty vector group (Figures 3J,K). Similarly, real-time monitoring migration/invasion assays indicated that knockdown of integrin α3 expression could impair the migration and invasion of CC cells (Figures 3L,M), whereas overexpression of integrin α3 could increase the migration and invasion of CC cells (Figures 3N,O).

### Integrin α3 Regulates Focal Adhesion Formation and Promotes the Migration and Invasion of Cervical Cancer Cells via Activation of Focal Adhesion Kinase

**Figure 4:**
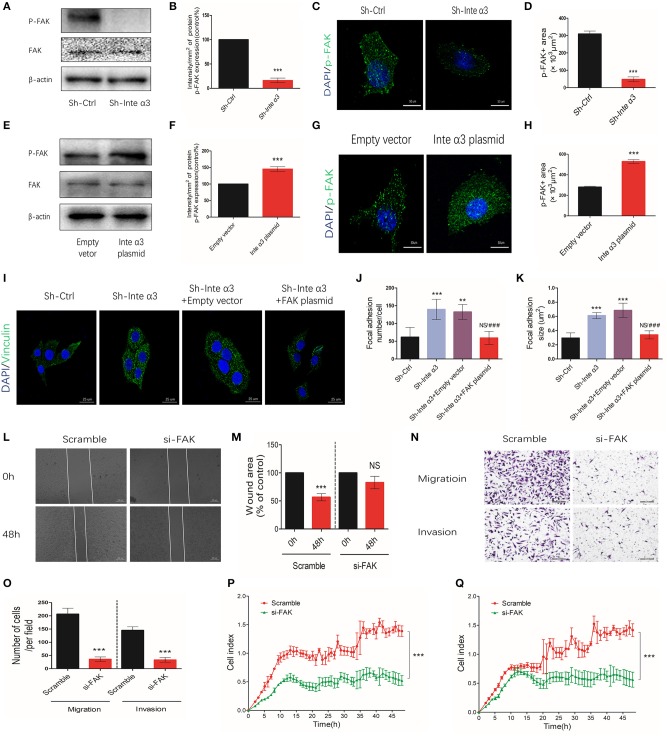
Integrin α3 impairs focal adhesion formation by activating focal adhesion kinase (FAK). **(A,B)** Western blotting results of phosphor-Y397-FAK (p-FAK) after knocking down the expression of integrin α3 using shRNA *in vitro* and quantification of the intensity of p-FAK protein. **(C,D)** After knocking down integrin α3, immunofluorescence was applied to detect the expression levels of p-FAK and quantification of the p-FAK-positive area. **(E,F)** Western blotting results of p-FAK after overexpressing of integrin α3 and quantification of the intensity of p-FAK protein. **(G,H)** Immunofluorescence results of p-FAK after overexpressing integrin α3 and quantification of the p-FAK-positive area. **(I)** Focal adhesion formation was investigated through vinculin immunofluorescence in the following four groups: Sh-Ctrl, Sh-Inte α3, Sh-Inte α+empty vector, and Sh-Inte α3+FAK plasmid. **(J,K)** Quantification of focal adhesion (number and size) in **(I)**. **(L)** The wound healing assay was applied to explore the migration of cervical cancer cells in Scramble and si-FAK groups, and the quantification is presented in **(M)**. **(N)** Transwell migration and invasion assays were performed in the two groups. **(O)** Quantification of migration and invasion (cell number) per field. **(P,Q)** Results related to cell migration and invasion using real-time migration/invasion monitoring. All experiments were repeated thrice with consistent results, and the representative images are shown. ^**^P < 0.01; ^***^P < 0.001; ^*###*^*P* < 0.001 vs. Sh-Inte α3+empty vector.

As mentioned in the *Introduction*, integrins could regulate cell focal adhesion formation and participate in cell migration and invasion processes. We found that the expression of phospho-Y397-FAK was significantly decreased after knocking down integrin α3, as shown by the western blotting (Figures 4A,B) and immunofluorescence results (Figures 4C,D). Inversely, overexpression of integrin α3 could lead to more phosphor-Y397-FAK indicated by increased western blotting intensity (Figures 4E,F) and p-FAK-positive areas (Figures 4G,H). At the resting status, focal adhesion formation was not activated in the control group, indicating smaller and less focal adhesion (Sh-Ctrl group). Treatment with integrin α3 shRNA activated focal adhesion formation, indicating larger size, and more numbers of focal adhesion locally. In addition, this effect would be diminished (Figures 4I–K) following treatment of cells with the FAK plasmid to overexpress FAK (efficiency shown in Figures S1N–P).

**Figure 5:**
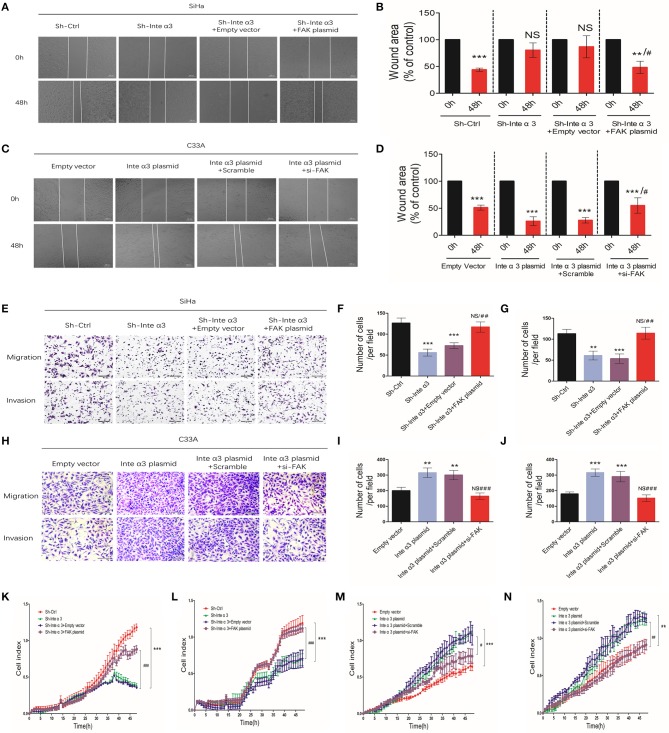
Integrin α3 promotes the migration and invasion of cells via focal adhesion kinase (FAK). **(A)** The wound healing assay was applied in SiHa cell to explore the migration of cervical cancer cells in the four groups—Sh-Ctrl, Sh-Inte α3, Sh-Inte α+empty vector, and Sh-Inte α3+FAK plasmid—and the quantification is presented in **(B)**. **(C)** The wound healing assay was applied in C33A cell in the four groups—empty vector, Inte α3 plasmid, Inte α3 plasmid+Scramble, and Inte α3 plasmid+si-FAK—and the quantification is presented in **(D)**. **(E)** Transwell migration and invasion assays in SiHa cells were performed in the four groups in **(A)**, and corresponding quantification of migration and invasion cells is shown in **(F,G)**. **(H)** Transwell migration and invasion assays in C33A cells were performed in the four groups in **(C)**, and corresponding quantification of migration and invasion cells is shown in **(I,J)**. **(K,L)** Results related to cell migration and invasion using real-time migration/invasion monitoring in SiHa cell in the aforementioned groups in **(A)**. **(M,N)** Results related to cell migration and invasion using real-time migration/invasion monitoring in C33A cell in the aforementioned groups in **(C)**. ^**^P < 0.01; ^***^P < 0.001; ^#^*P* < 0.05 vs. corresponding group (e.g., Sh-Inte α+empty vector vs. Sh-Inte α3+FAK plasmid); ^##^*P* < 0.01; ^###^*P* < 0.01.

Furthermore, we wished to determine whether integrin α3 could regulate the migration and invasion of cells through FAK. In normal condition, fewer wound healing areas (Figures 4L,M) and less migrated or invaded cells (Figures 4N,O) were observed after knocking down FAK with FAK siRNA (efficiency shown in Figures S1F,G). Similarly, real-time monitoring migration/invasion assays indicated that knockdown of FAK expression could impair the migration and invasion of CC cells (Figures 4P,Q). The results of the wound healing assays revealed more wound healing areas in the Sh- Integrin α3+FAK plasmid group compared with the corresponding control group, demonstrating that overexpression of FAK could reverse the impairing effect of integrin α3 on the migration of SiHa cells (Figures 5A,B). Conversely, another rescue assay indicated that knocking down of FAK could reverse the upregulation effect of integrin α3 on the migration of C33A cells (Figures 5C,D). Similar results were also observed through the transwell migration/invasion assays (Figures 5E–J). The results of the real-time monitoring migration/invasion assays corroborated these findings (Figures 5K–N).

Collectively, these data showed that integrin α3 regulates focal adhesion formation and promotes the migration and invasion of CC cells via activation of FAK, partly revealing the regulatory mechanism of integrin α3 involved in CC metastasis.

### Integrin α3 Activates Focal Adhesion Kinase via Activation of the c-Src/Erk Cascade

**Figure 6:**
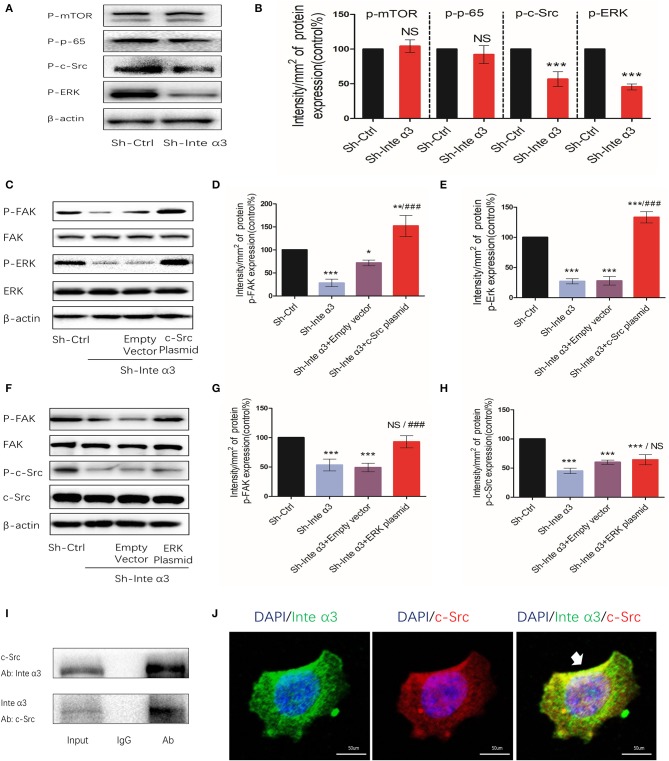
Integrin α3 activates focal adhesion kinase (FAK) via the c-Src/extracellular signal-regulated protein kinase (ERK) signaling pathway. **(A)** Phosphorylated mammalian target of rapamycin (mTOR), p-65, c-Src, and ERK were examined through western blotting in the Sh-Ctrl and Sh-Inte α groups; the corresponding quantification of the protein intensity is shown in **(B)**. **(C)** The levels of phosphorylated FAK and ERK, as well as total FAK, ERK, and β-actin, were determined via western blotting among the Sh-Ctrl, Sh-Inte α3, Sh-Inte α3+empty vector, and Sh-Inte α3+c-Src plasmid groups; the corresponding quantification of p-FAK and p-c-Src protein intensity is presented in **(D,E)**. **(F)** Among the Sh-Ctrl, Sh-Inte α3, Sh-Inte α3+empty vector, and Sh-Inte α3+ERK plasmid groups, the levels of phosphorylated FAK and c-Src, as well as total FAK, c-Src, and β-actin, were examined through western blotting; the quantification of p-FAK and p-ERK protein intensity is shown in **(G,H)**. **(I)** Representative image of co-immunoprecipitation using anti-c-Src or anti-integrin α3, and subsequent detection of the other protein. **(J)** Representative immunofluorescence images showing colocalizations between integrin α3 and c-Src. Nuclei were stained with 4′-6-diamidino-2-phenylindole (DAPI) (blue), anti-c-Src linked to Alexa Fluor (red), anti-integrin α3 linked to fluorescein isothiocyanate (FITC) (green); original magnification: ×200. All experiments were repeated thrice with consistent results, and the representative images are shown. ^*^P < 0.05 vs. control group; ^**^P < 0.01; ^***^P < 0.001; ^###^*P* < 0.01.

Some signaling intermediates were tested in the Sh-Ctrl and Sh-Inte α3 groups to investigate the signaling pathways involved in the regulatory effect of integrins. Phosphor-mTOR and phosphor-P-65, which are important parts of the mTOR and nuclear factor-κB signaling pathways, respectively, were not significantly changed, whereas decreased expression of phosphor-c-Src and phosphor-ERK was noted (Figures 6A,B). This result suggests that the c-Src and ERK pathways may be involved in this regulatory process.

Indeed, overexpression of c-Src and ERK by applying their plasmids led to increased expression levels of phosphor-FAK (efficiency shown in Figures S1H–M). These results indicated that c-Src and ERK may participate to some extent in the regulation of FAK activation by integrin α3 (Figures 6C,F). Moreover, following overexpression of c-Src or ERK after knockdown of integrin α3, phosphor-FAK could reverse to higher expression compared with the control group in this rescue assay. After overexpression of c-Src, the levels of phosphor-ERK were increased. In contrast, overexpression of ERK did not lead to a change in the levels of phosphor-c-Src protein (Figures 6C–H). Taken together, the results demonstrated that the c-Src pathway was upstream of the ERK signaling pathway in this regulatory process.

In the quiescent state, an interaction between integrin α3 and c-Src was observed, as demonstrated by the immunoprecipitation assay (Figure 6I). This was further verified through the immunofluorescence analysis (Figure 6J), indicating that c-Src could interact with integrin α3 and was firstly triggered in this c-Src/ERK/FAK cascade.

### Integrin α3 Is Related to Microvascular Density and Induces Angiogenesis via Matrix Metalloproteinase-9

**Figure 7:**
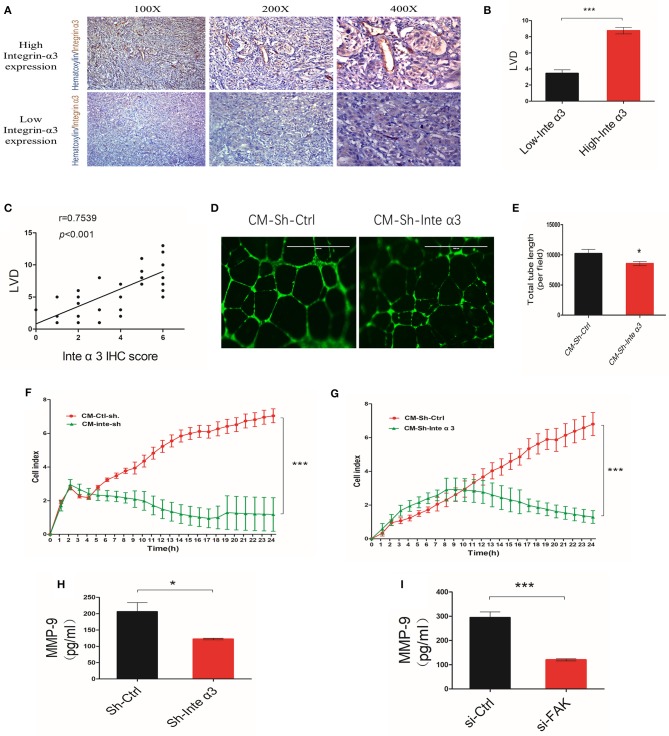
Integrin α3 promotes angiogenesis by secreting metalloproteinase-9 (MMP-9) *in vitro*. **(A,B)** Immunohistochemistry (IHC) staining and corresponding quantification of CD34 in the high/low integrin α3 expression groups in cervical cancer (CC) tissue. Original magnification: ×100, ×200, and ×400. **(C)** Correlation of IHC score and microvascular density. The correlation coefficient is shown in the panel. **(D)** The human umbilical vein endothelial cell (HUVEC) tube formation assay was applied to explore the effect of integrin α3 on angiogenesis. **(E)** Quantification of the total tube length per field is shown in **(D)**. **(F,G)** Real-time migration/invasion monitoring was used for the detection of migration and invasion of HUVECs treated with the cell culture medium from the Sh-Ctrl and Sh-Inte α3 groups. **(H,I)** ELISA for MMP-9 was performed after knocking down integrin α3 and focal adhesion kinase (FAK). All experiments were repeated thrice with consistent results, and the representative images are shown. ^*^P < 0.05 vs. control group; ^***^P < 0.001.

An astonishing aspect of the effect of integrins is its relationship with angiogenesis. Thus, we divided 40 CC tissue samples into high and low integrin α3 expression groups according to the aforementioned IHC scoring. Microvascular density (MVD) was also evaluated in these samples through CD-34 IHC staining, showing that the high expression group presented more MVD than had the low expression group (Figures 7A,B). Moreover, the correlation analysis demonstrated that integrin α3 expression was related to MVD (Figure 7C), revealing the likely involvement of integrin α3 in CC angiogenesis.

HUVEC tube formation assays were performed to further confirm its role in CC angiogenesis. The total tube length was remarkably reduced in the Sh-Inte α3 group vs. the control group (Figures 7D,E). Interestingly, the culture medium from the Sh-Inte α3 group could negatively regulate the growth and migration of HUVECs (Figures 7F,G), emphasizing its effect on angiogenesis.

Because the culture medium from the Sh-Inte α3 group could impair angiogenesis, we subsequently intended to identify which angiogenic factor in the medium was affected. MMP-9 enzyme-linked immunosorbent assay analysis revealed that secretion of MMP-9 was indeed decreased in the culture medium from the Sh-Inte α3 group compared with the control group (Figure 7H). In addition, knocking down of FAK (knockdown efficiency is shown in Figures S1F,G) with FAK siRNA led to a reduction in the secretion of MMP-9 (Figure 7I).

Collectively, these data demonstrated that integrin α3 is related to MVD in CC tissues and could induce angiogenesis *in vitro* by secreting MMP-9.

### Integrin α3 Promotes Cervical Cancer Metastasis *in vivo*

**Figure 8:**
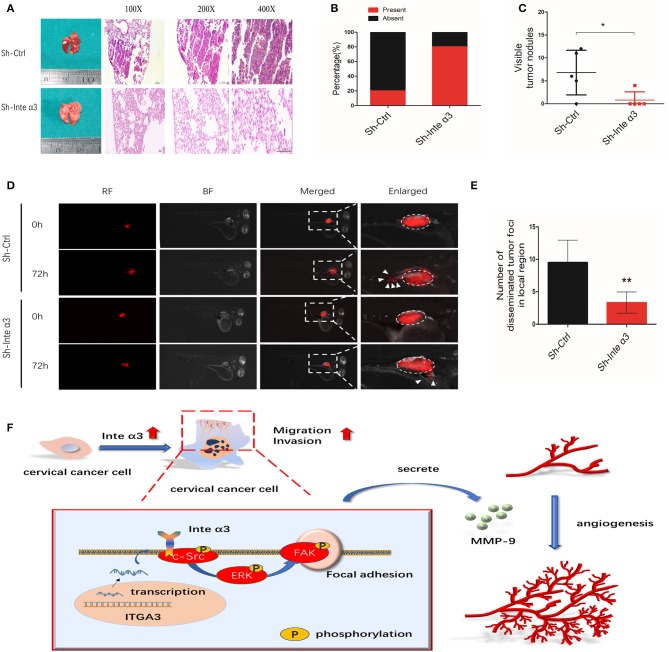
Integrin α3 promotes metastasis *in vivo*; a proposed schematic representation of the mechanism. **(A)** Typical H&E images of pulmonary metastatic foci in the Sh-Ctrl group and normal pulmonary tissues in the Sh-Inte α3 group are shown. Original magnification: ×100, ×200, and ×400. **(B)** Percentages of mice with or without metastatic foci in their lungs in the aforementioned two groups (*n* = 5). **(C)** Visible tumor nodules in the lungs were calculated in these two groups. **(D)** The zebrafish tumor migration model was applied to explore the migration ability of cells in these two groups. **(E)** Quantification of the number of disseminated foci in the local region of **(D)**. **(F)** Proposed schematic representation of the mechanism demonstrating that integrin α3 activates the c-Src/Erk/focal adhesion kinase (FAK) signaling pathway and promotes cervical cancer metastasis and angiogenesis. ^*^P < 0.05 vs. control group; ^**^P < 0.01.

The effect of integrin α3 expression on the migration and invasion of CC cells was verified using the aforementioned *in vitro* assays. We firstly established a stable knockdown of integrin α3 in SiHa cells using a lentivirus (Figures S1A–C), and we subsequently adopted a xenograft mouse metastatic model in female BALB/c nude mice. After 6 weeks, visible nodules in the mice lungs were counted, and H&E staining of their slices was performed to evaluate the metastatic foci. A higher ratio of metastatic lungs and more visible nodules were observed in the control group than in the Sh-Inte α3 group (Figures 8A–C).

In addition, a zebrafish model of CC migration was established to further confirm the effect of integrin α3 on CC metastasis. A reduced number of disseminated tumor foci in the local region was counted in the Sh-Inte α3 group vs. the control group (Figures 8D,E).

## Discussion

Although screening and vaccination against human papillomavirus have greatly reduced the incidence of CC, CC metastasis remains the major problem in this setting. The 5-year survival rate for patients with metastatic CC is tremendously lower than that reported for patients with localized CC (16.5 vs. 91.5%, respectively) (18). The treatment options for metastatic CC are limited. However, advances in therapies have been achieved owing to clinical trials investigating the optimal approach to prolonging OS and DFS in those patients (19). Unfortunately, the mechanism of CC metastasis remains largely unclear, posing a challenge to gynecologists and oncologists in terms of defining and discovering potential prognostic biomarkers or treatment targets. Additional research is warranted to prolong the survival of patients with CC (20). Treatment with an anti-angiogenesis drug (e.g., bevacizumab) combined with traditional chemotherapy (e.g., cisplatin and paclitaxel) is a potential option (21). Although favorable outcomes have been noted, additional potential diagnostic markers or therapeutic targets are warranted. In this aspect, the present study revealed that integrin α3 could predict poor outcome in patients with CC and promote metastasis and angiogenesis via activation of the c-Src/Erk/FAK signaling pathway, which may shed light on the diagnosis and therapy of CC metastasis.

Members of the integrin family (e.g., α3β1, α6β1, α6β4, α2β1, α5β1, and α1β1) are aberrantly expressed and involved in tumorigenesis and metastasis in various cancer cells (22). Among these molecules, integrin α3 was found to be expressed in several types of cancer; however, it plays different roles owing to its oncogenic or antitumor activity. For instance, in pancreatic duct adenocarcinoma cells, integrin α3 interacts with laminin-332 to maintain cancer-associated-fibroblasts, thus supporting cancer invasion (23). In contrast, in prostate cancer, integrin α3 inhibits cancer metastasis (7). Those studies suggested that integrin α3 is tissue specific and cancer specific. Thus, its role in different types of cancer could be varied, indicating that exploration of its expression and specific mechanism is necessary. In CC, integrin α3β1 was expressed in 20 cases of invasive CC (24). To the best of our knowledge, the present study is the first to demonstrate that integrin α3 could predict poor survival and be related to metastasis and angiogenesis in CC.

Interestingly, integrins are receptors that facilitate cell–extracellular matrix adhesion and their relationship with angiogenesis, which are essential for tumor progression (25). Indeed, integrins could modulate cell mobility and adhesion movement (26). Additionally, integrins were reported to play vital role in sensing, integrating, and distributing angiogenesis-related cellular events or pathways (27). Therefore, in this study, we mainly investigated the effect of integrin α3 on CC cell migration, invasion, and angiogenesis.

Focal adhesion formation has been associated with cancer metastasis (28). Activation of FAK is essential for focal adhesion formation and cell motility (28). In CC, FAK was shown to promote CC tumorigenesis (29) and participate in the migration and invasion of CC cells (30). The activated integrin α3 leads to activation of FAK (31). Consistently, our data also clarified that knockdown of integrin α3 expression decreased the level of FAK phosphorylation. Overexpression of FAK following knockdown of integrin α3 rescued the inhibitory effect of integrin α3 on the migration/invasion of CC cells and the enhancement of focal adhesion. This analysis provided new evidence concerning the regulation of FAK and focal adhesion formation by integrin α3.

Regarding the mechanism involved in this process, we identified that integrin α3 can interact with c-Src, which serves as the trigger of the signaling pathway, activating the ERK/FAK cascade. We initially examined four potential signaling pathways, namely, mTOR, nuclear factor-κB, c-Src, and ERK. The results showed that only c-Src and ERK had been modulated after knockdown of integrin α3. Inhibition of integrin α3 reduced the level of c-Src phosphorylation (32), which was supportive of our data. We also provided evidence that integrin α3 interacted with c-Src in CC cells to trigger the signaling pathway. ERK was shown to be downstream of the c-Src pathway, consistent with the results of another research study (33). In addition, rescue assays demonstrated that overexpression of c-Src or ERK increased the expression of phosphor-FAK. Nevertheless, the mechanism through which activation of c-Src/ERK leads to upregulation of phosphor-FAK warrants further investigation in future studies.

Another notable aspect of integrin α3 is its regulation of angiogenesis. In this study, we also investigated its role on CC angiogenesis. Firstly, the expression of integrin α3 was correlated with MVD. Secondly, our findings demonstrated that inhibition of integrin α3 affected endothelial cell tube formation, proliferation, and migration, which are the initial steps of the cancer neovascularization process (34). Angiogenic factors (e.g., MMP-2, MMP-9, vascular endothelial growth factor, and platelet-derived growth factor family) may be consequently affected by the regulatory effects of integrins (35). MMP-9 is the key direct regulator of cancer angiogenesis and affects neovascularization in numerous manners (36). Additionally, integrin α3 was found to regulate the expression of the MMP-9 gene by affecting mRNA stability (37, 38). Similarly, we found that integrin α3 promotes angiogenesis by secreting MMP-9. Furthermore, knockdown of FAK also reduced the secretion of MMP-9. Thus, we provided evidence that integrin α3 induces angiogenesis in CC cells by activating the FAK/MMP-9 axis.

In conclusion, the results of the present study implied that integrin α3 interacts with c-Src and recruits the ERK/FAK cascade, leading to the impairment of focal adhesion formation. This effect endows CC cells with potentiated migratory and invasive ability and induces cancer angiogenesis via secretion of MMP-9. According to our findings, a schematic representation of the mechanism involved in this process (Figure 8F) was proposed. Our findings may shed light on the mechanism of CC metastasis and angiogenesis, highlighting integrin α3 as a candidate prognostic biomarker and therapeutic target in patients with CC.

## Data Availability Statement

All datasets generated for this study are included in the article/Supplementary Material.

## Ethics Statement

The studies involving human participants were reviewed and approved by The Ethics Committee of the First Affiliated Hospital of Sun Yat-sen University. The patients/participants provided their written informed consent to participate in this study. The animal study was reviewed and approved by The Animal Ethical and Welfare Committee of Sun Yat-sen University.

## Author Contributions

This work was designed by QD under the guidance of SY and JL. QD mainly performed the experiments and wrote the manuscript. WW, TL, and CS provided technical support and assisted with the *in vitro* assays. JH, YL, SQ, YC, and PL collected the clinical patient samples. SY and JL contributed to the revision of the manuscript.

### Conflict of Interest

The authors declare that the research was conducted in the absence of any commercial or financial relationships that could be construed as a potential conflict of interest.
