# Supplementary material for: High Expression of Integrin α3 Predicts Poor Prognosis and Promotes Tumor Metastasis and Angiogenesis by Activating the c-Src/Extracellular Signal-Regulated Protein Kinase/Focal Adhesion Kinase Signaling Pathway in Cervical Cancer
Source: Front Oncol. 2020 Feb 14;10:36. doi: 10.3389/fonc.2020.00036 (PMC7033469; doi:10.3389/fonc.2020.00036)
Supplement: Supplementary file 1 [file Image_1.pdf]

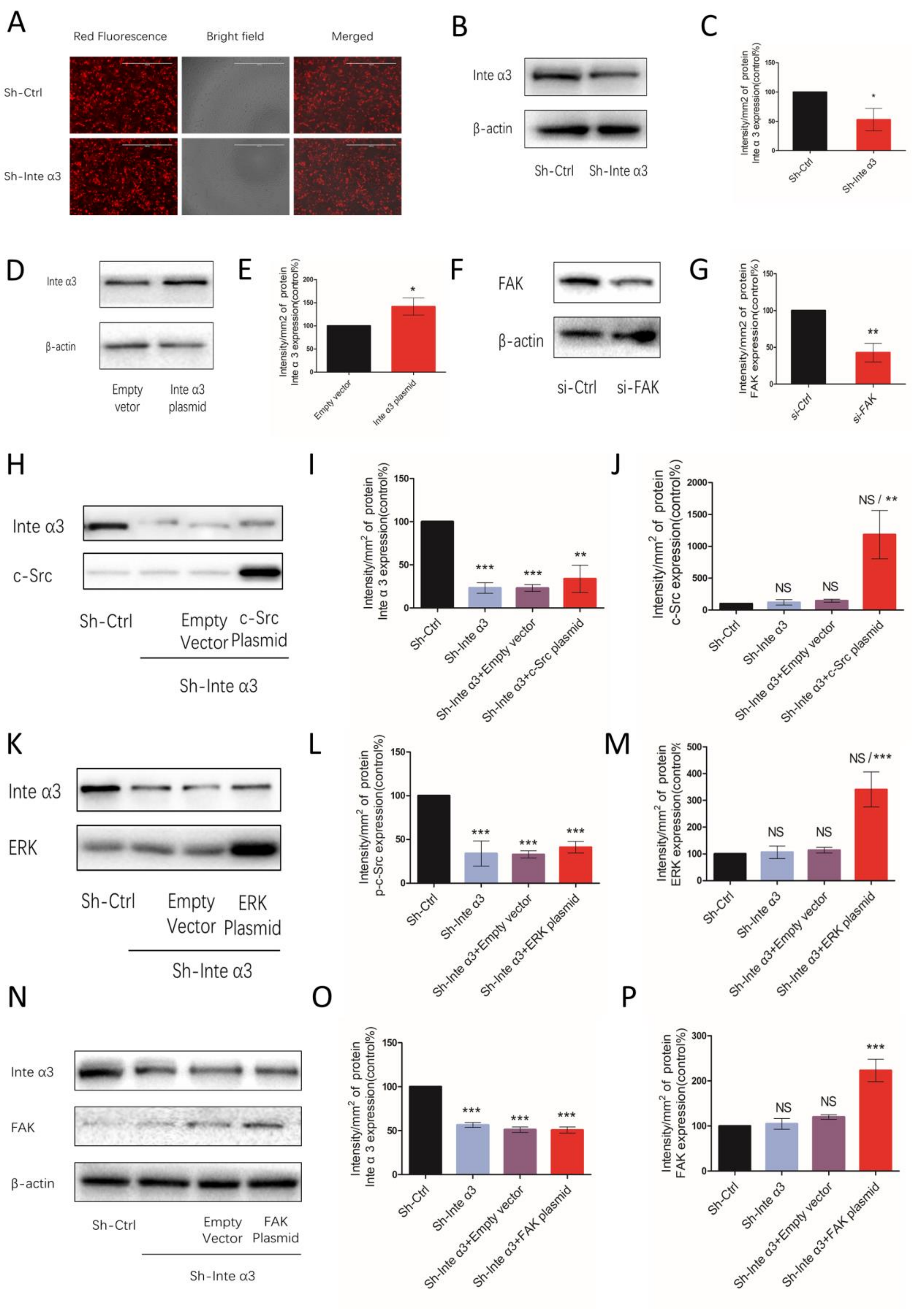
A
Red Fluorescence
Bright field
Merged
Sh-Ctrl
Sh-Inte α3
B
Inte α3
β-actin
Sh-Ctrl
Sh-Inte α3
C
D
Inte α3
β-actin
Empty vetor
Inte α3 plasmid
E
F
FAK
β-actin
si-Ctrl
si-FAK
G
H
Inte α3
c-Src
Sh-Ctrl
Empty Vector
c-Src Plasmid
Sh-Inte α3
I
J
NS / **
K
Inte α3
ERK
Sh-Ctrl
Empty Vector
ERK Plasmid
Sh-Inte α3
L
M
NS / ***
N
Inte α3
FAK
β-actin
Sh-Ctrl
Empty Vector
FAK Plasmid
Sh-Inte α3
O
P

Figure S1. **Efficiency of shRNA/siRNA/plasmid in this study. (A)** Red fluorescence and bright field images of SiHa cell treated with ITGA3 lentivirus and corresponding control lentivirus, showing lentivirus transfection rate. Original magnification: ×100; **(B)** Western blotting images showed the integrin α3 level change after treated with lenti-ITGA3 lentivirus. **(C)** Quantification of protein intensity in (C); **(D)** Western blotting images showed the integrin α3 level change after treated with integrin α3 plasmid. **(E)** Quantification of protein intensity in (D); **(F)** Western blotting images showed the FAK level change after treated with FAK siRNA. **(G)** Quantification of protein intensity in (F); **(H)** Integrin α3 and c-Src expression were showed by western blotting among groups Sh-Ctrl, Sh-Inte α3， Sh-Inte α3+Empty vector and Sh-Inte α3+c-Src plasmid, responding quantification of integrin α3 and c-Src protein intensity were presented in **(I)** and **(J)**; **(K)** Integrin α3 and ERK expression were showed by western blotting among groups Sh-Ctrl, Sh-Inte α3， Sh-Inte α3+Empty vector and Sh-Inte α3+ERK plasmid, responding quantification of integrin α3 and ERK protein intensity were presented in **(L)** and **(M)**; **(N)** Integrin α3 ,FAK, β -actin expression were showed by western blotting among groups Sh-Ctrl, Sh-Inte α3， Sh-Inte α3+Empty vector and Sh-Inte α3+FAK plasmid, responding quantification of integrin α3 and FAK protein intensity were presented in **(O)** and **(P)**;

* = P<0.05 vs. corresponding control; ** = P<0.01; ***= P<0.001; #= P<0.05 vs. corresponding group (e.g. Sh-Inte α+empty vector vs Sh-Inte α3+FAK plasmid), ##= P<0.01, ###= P<0.01; All the experiments were repeated three times with consistent results, and the representative images are shown.
